# Alcohol Interactions with Psychostimulants: An Overview of Animal and Human Studies

**DOI:** 10.4172/2155-6105.1000281

**Published:** 2016-06-11

**Authors:** Yusuf S. Althobaiti, Youssef Sari

**Affiliations:** University of Toledo, College of Pharmacy and Pharmaceutical Sciences, Department of Pharmacology and Experimental Therapeutics, Toledo, OH, USA

**Keywords:** Alcohol, Ethanol, Methamphetamine, Nicotine, Cocaine, Alcohol interaction, 3,4-Methylenedioxymethamphetainen

## Abstract

Alcohol consumption with psychostimulants is very common among drug addicts. There is little known about the possible pharmacological interactions between alcohol and psychostimulants. Among most commonly co-abused psychostimulants with alcohol are methamphetamine, cocaine, 3,4-methylenedioxymethamphetaminen, and nicotine. Co-abuse of alcohol with psychostimulants can lead to several neurophysiological dysfunctions such as decrease in brain antioxidant enzymes, disruption of learning and memory processes, cerebral hypo-perfusion, neurotransmitters depletion as well as potentiation of drug seeking behaviour. Moreover, co-abuse of alcohol and psychostimulants can lead to increase in heart rate, blood pressure, myocardial oxygen consumption and cellular stress, and the risk of developing different types of cancer. Co-abuse of alcohol with psychostimulants during pregnancy can lead to fetal brain abnormalities. Further studies are needed to investigate the pharmacokinetics, pharmacodynamics, and neurochemical changes on co-abuse of alcohol and psychostimulants.

## Introduction

Alcohol dependence is considered a major public health problem worldwide [1–6]. Alcohol can contribute to a significant number of disabilities due to psychological, medical, injury, or other detrimental effects [7]. These effects can be dramatically severe when alcohol is consumed with other drugs of abuse. Alcohol consumption with other drugs of abuse is very common among drug users. Different pharmacological mechanisms of interactions may occur when alcohol and other psychostimulants are co-abused. It is noteworthy that drugs of abuse have been shown to alter central brain reward circuitry, which can lead addicts to increase their alcohol intake for reward effects [8,9]. Alcohol use with other drugs of abuse has been reported to hinder decision making, thinking, and neurocognitive capabilities [10–15]. Moreover, recent studies confirmed that alcohol and other drugs of abuse are usually found in the blood of deceased or seriously injured drivers involved in traffic accidents caused by psychomotor function impairment [16–20].

We discussed here several findings related to alcohol interactions with psychostimulants. According to previous reports, alcohol is commonly abused with methamphetamine (METH), cocaine and marijuana [21]. Men have higher prevalence of co-abuse of alcohol and other drugs compared to women [22]. The prevalence of drugs of abuse has been shown to have a positive correlation with the level of alcohol intake [22]. We reviewed here the available literature regarding alcohol interactions with certain psychostimulants, including METH, cocaine, nicotine, and 3,4-methylenedioxymethamphetaminen (MDMA), according to animal experimental and clinical studies.

## Alcohol and METH co-abuse

METH abuse is an increasing health problem worldwide. According to the available data from national surveys between the years of 2002 and 2004, more than 16 million Americans over the age of 12 have used METH [23]. METH is a derivative of amphetamine with increased CNS activities and effects. METH can be abused by different routes such as inhalation, ingestion, or intravenous injection, with acute effects that can last for up to 24 h [24,25]. It is well known that METH can stimulate the release of monoamines such as dopamine and norepinephrine to produce euphoria and to increase alertness and libido [26–28]. METH abusers frequently use alcohol and have a higher risk of reaching alcohol intoxication level [29]. The prevalence of alcohol use disorder was found 75% higher among amphetamine dependent patients [30]. For example, a study reported that more than 60% of METH users in New York City reported abusing METH in combination with alcohol [31]. Recent study conducted on regular METH users showed that alcohol drinking increased the chances of METH use in same day by more than 4 folds [32]. Despite this evidence of high prevalence of METH and alcohol co-abuse, very few studies have investigated the effects of their co-abuse. A summary of possible effects of concurrent exposure to alcohol and METH is presented in Table 1.

**Figure 1:**
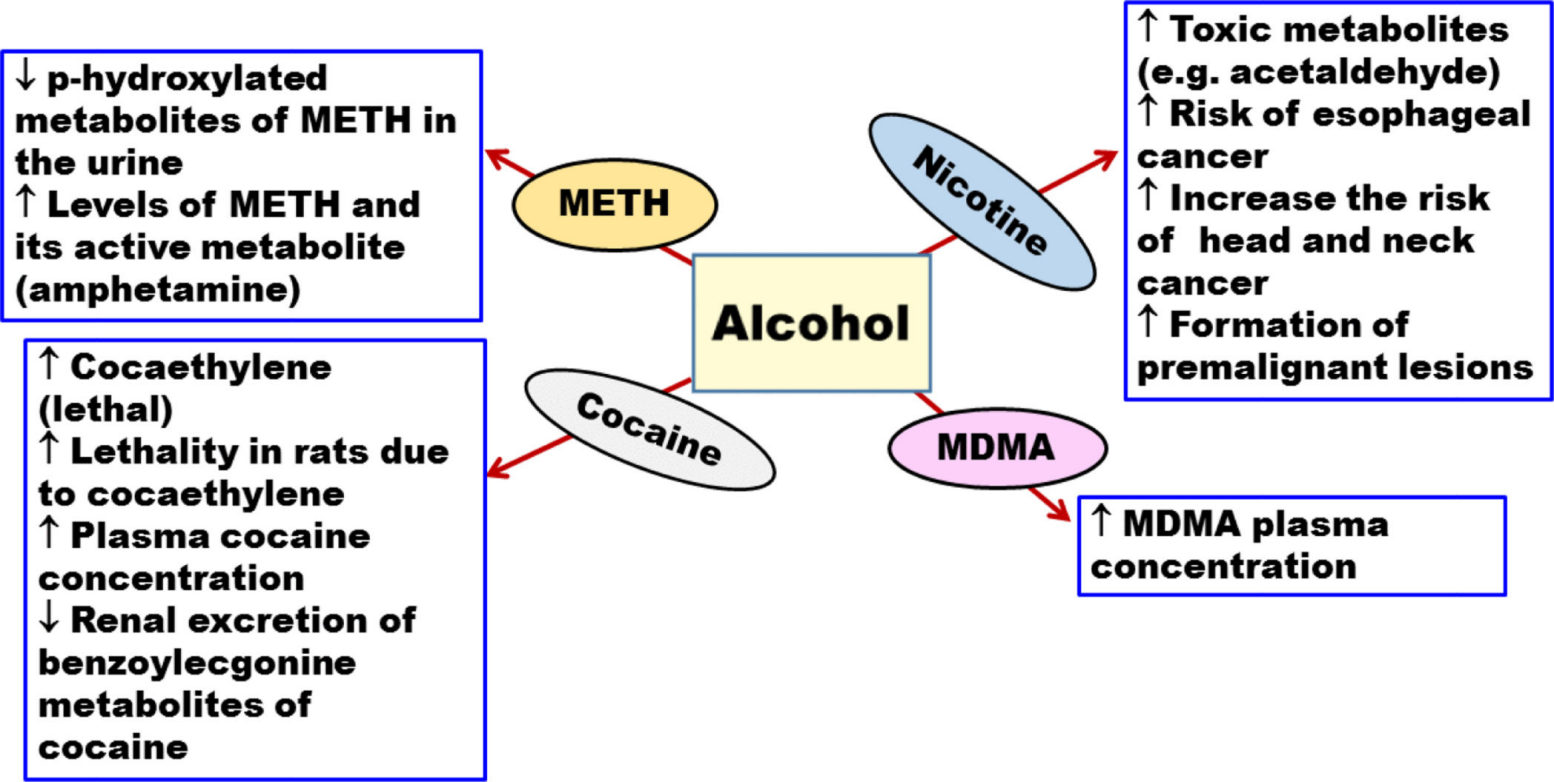
Effects of alcohol on the pharmacokinetics of methamphetamine(METH), 3,4-methylenedioxymethamphetaminen (MDMA), cocaine, and nicotine. (↑ increase or enhancement; ↓ decrease or deterioration).

Previous findings demonstrated that alcohol can decrease p-hydroxylated metabolites of METH in the urine of METH abusers, suggesting that alcohol may inhibit METH metabolism [33] (Table 1). This may lead to higher METH blood concentration, with an increase in its stimulating effects on brain and heart. Moreover, recent findings showed that alcohol increased the absorption and distribution of METH and its active metabolite, amphetamine, in several organs, including brain in rats and rabbits (Figure 1) [34,35]. A recent study compared the acute effects of alcohol, METH, and their combination on mood, performance, and physiological behaviours of nine adult males [36]. This study showed that when alcohol and METH were co-self-administered, a greater increase in heart rate, euphoria, and lower detrimental effects on sleep and performance were observed compared to each drug self-administered alone. This may explain why METH abusers tend to consume high level of alcohol [36]. These findings raise an alarming concern of METH and alcohol co-abuse because METH might mask the signs of alcohol intoxication, such as sedation and compensated performance, allowing abusers to consume more alcohol with risk of developing alcohol toxicity.

**Figure 2:**
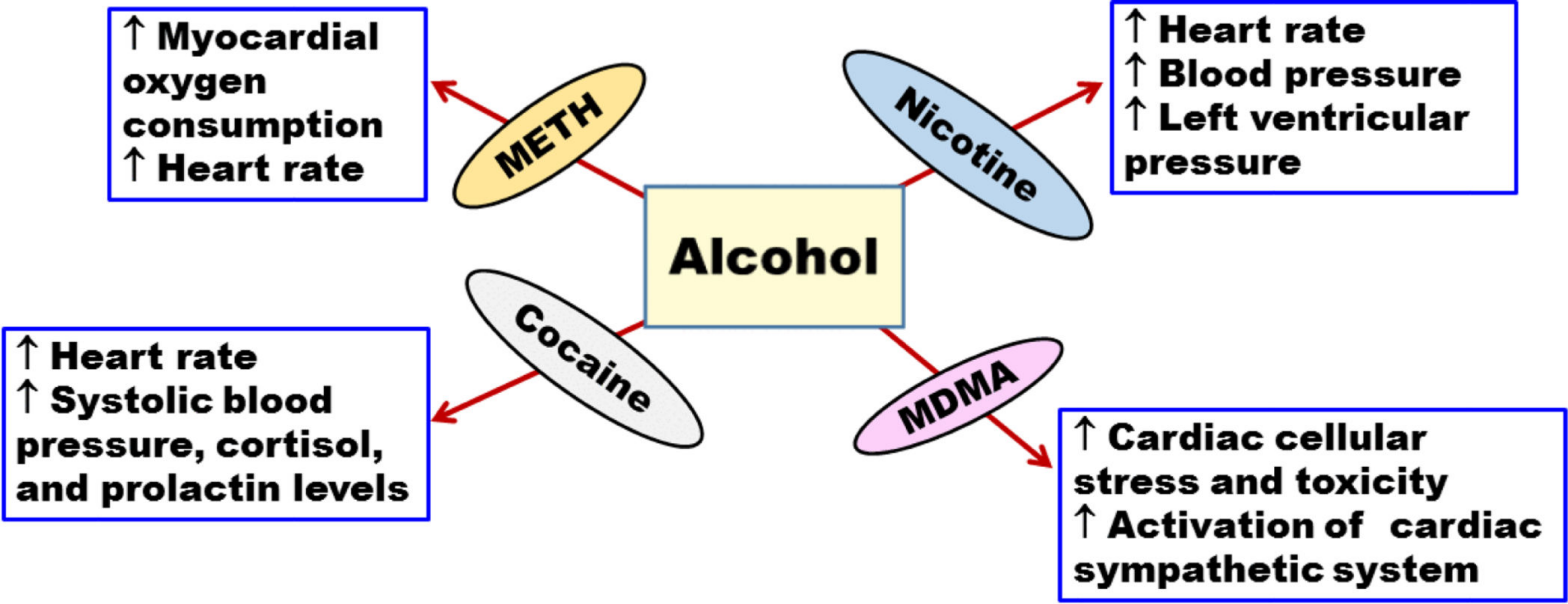
Effects of alcohol interactions with methamphetamine (METH),3,4-methylenedioxymethamphetaminen (MDMA), cocaine and nicotine on cardiovascular system, (↑ increase or enhancement; ↓ decrease or deterioration).

**Figure 3:**
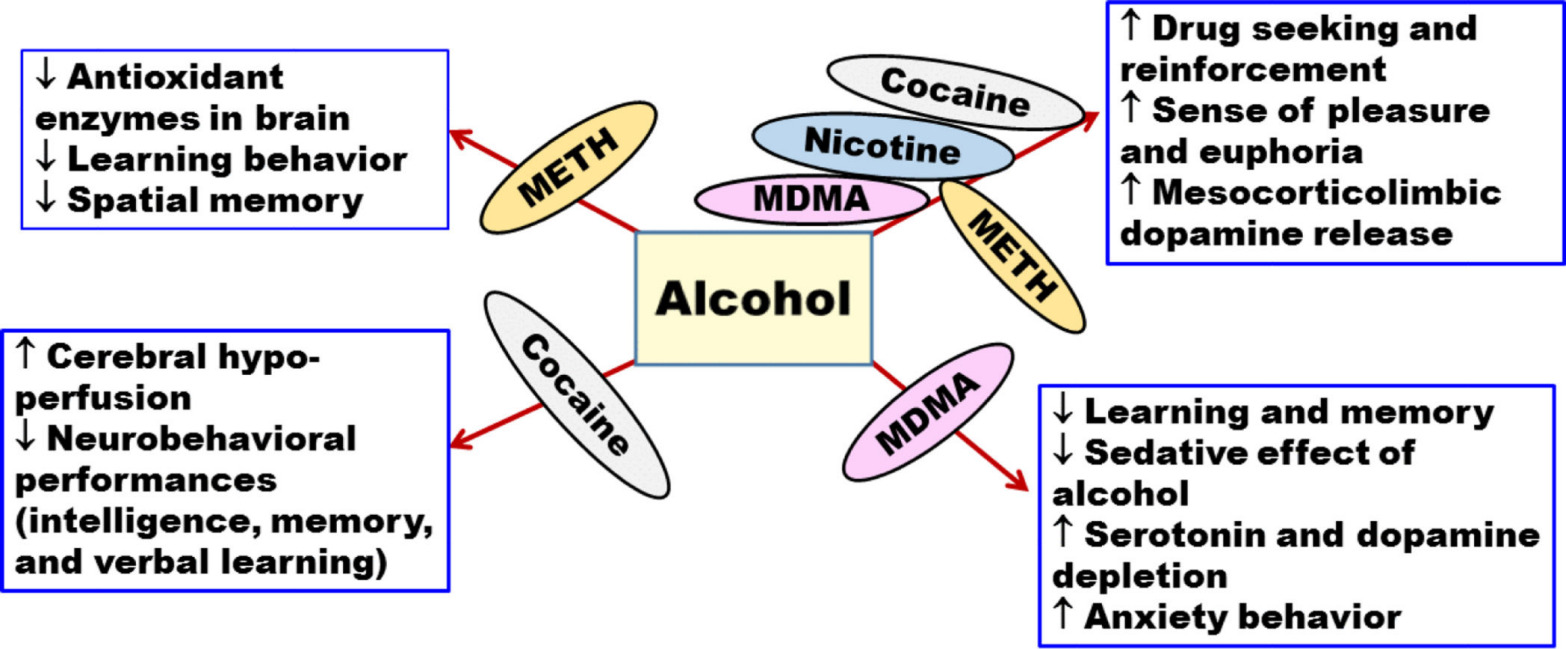
Effects of alcohol interactions with methamphetamine (METH),3,4-methylenedioxymethamphetaminen (MDMA), cocaine, and nicotine on central nervous system. (↑ increase or enhancement; ↓ decrease or deterioration).

A double blind study was conducted on eight alcohol and METH abusers [37]. The abusers were found to have high myocardial oxygen consumption and increased heart rate (Figure 2). In this study, the pharmacokinetics of METH did not change significantly, which is possibly due to the limited number of subjects recruited in this study. However, further testing should be done on more subjects to reach more conclusive evidence of the effect of alcohol on METH pharmacokinetics [37]. A recent clinical study conducted on nine volunteers showed an increase in heart rate [36]. Furthermore, findings showed that concurrent consumption of METH and alcohol disrupted learning and discriminating behaviour compared to METH self-administered alone in rats [38]. However, this study did not focus on the effects of alcohol alone which may hinder the conclusion that METH and alcohol co-abuse may disturb the performance compared to METH administered alone. A recent study conducted on rats revealed that concurrent intake of METH and alcohol can lead to synergistic effect in impairment of spatial memory compared to METH administered alone [39]. Interestingly, alcohol administered alone did not cause any changes in spatial memory suggesting the synergistic effects of both drugs on memory. Moreover, this study showed that although alcohol or METH administered alone can induce oxidative stress and impairments in antioxidant enzymes in rats hippocampus, co-abuse of METH and alcohol can cause synergistic effect in impairment and oxidative stress compared to drug administered alone (Figure 3) [39].

**Figure 4:**
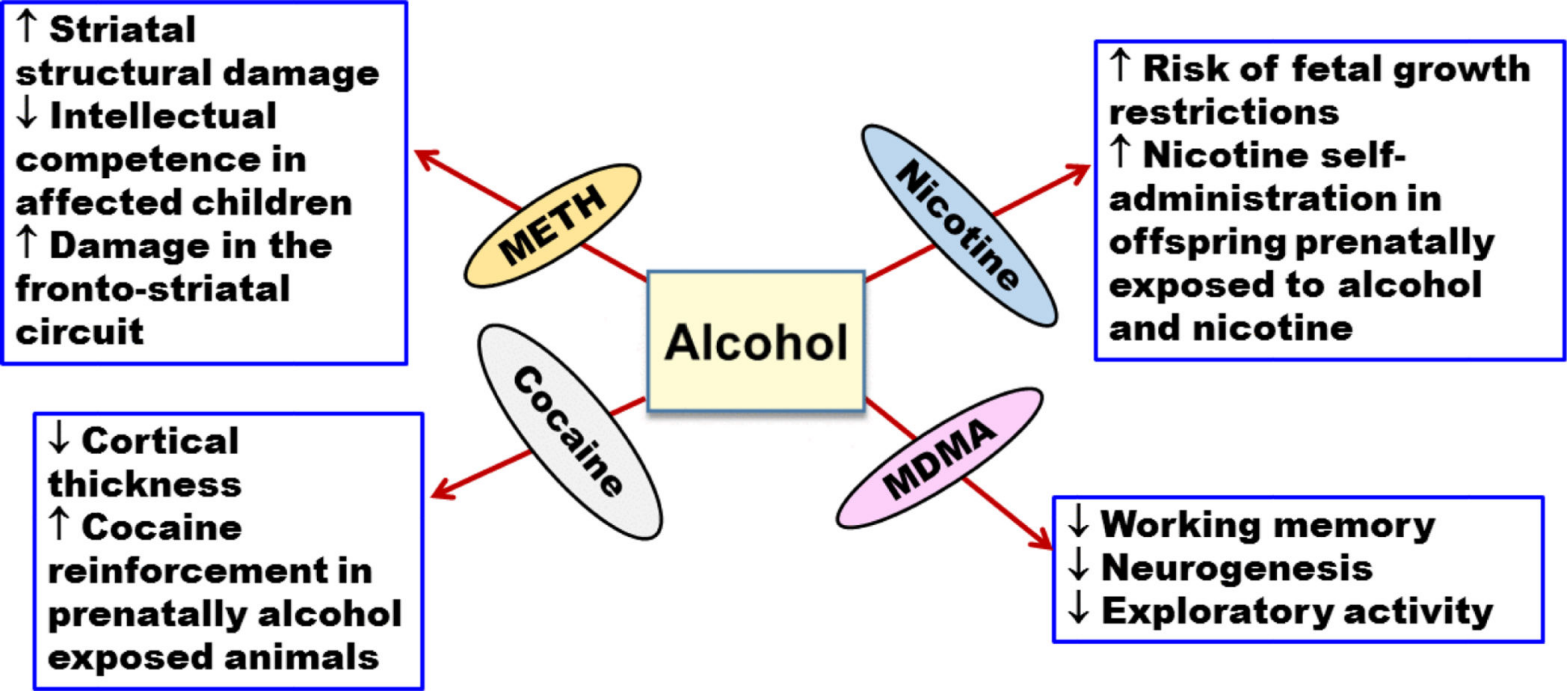
Effects of prenatal exposure to alcohol and methamphetamine(METH),3,4-methylenedioxymethamphetaminen (MDMA), cocaine, and nicotine. (↑ increase or enhancement; ↓ decrease or deterioration).

Concurrent exposure to METH and alcohol has also been observed in pregnant women. Indeed, a study demonstrated that more than 40% of pregnant women who abused METH reported using alcohol during their pregnancy [40]. Another study was conducted on 61 participants; 13 of them were exposed prenatally to alcohol, 21 were exposed prenatally to METH, and the remaining 27 control participants were not prenatally exposed to either alcohol or METH [41]. Of the 21 participants in METH group, 18 children were also exposed to alcohol during their fetal life. The results of this study suggest that prenatal exposure to METH and alcohol can cause synergistic striatal structural damage than prenatal exposure to alcohol alone. Damage to the striatal brain region hinders the overall intellectual competence of the affected children [41]. In addition, in a study that found common damage in the fronto-striatal circuit of the prenatally METH exposed group, 15 out of 19 children were exposed to alcohol and METH prenatally (Figure 4) [42]. It is important to note, however, that these studies could not precisely predict the dosage, frequency, and duration of METH or alcohol exposure during pregnancy, which may hamper our understanding of the pharmacological and neuropathological basis of drugs exposure and their interactions. Preclinical studies are warranted to show the risk of concurrent exposure of METH and alcohol during different stages of pregnancy, which may provide information about the deteriorating effects of prenatal exposure of METH and alcohol.

## Alcohol and cocaine co-abuse

Cocaine can produce different effects on the human body; these effects can last from minutes to hours, based on the route through which cocaine was administered into the body [43]. In the brain, cocaine can affect the reward circuitry by modulating dopamine neurotransmission [44], and acts by preventing the reuptake of dopamine from the synaptic cleft, which leads to prolongation of the pleasurable effects of dopamine [44,45]. Cocaine can produce euphoria, alertness, dependence and tolerance as well as cardiovascular changes [46–50]. Tolerance makes cocaine users increase dosage each time to reach the same level of euphoria that was reached on the first instance of taking the drug. Increasing the doses of cocaine can lead to its side effects and toxicity [51–54]. It is important to note that the prevalence of alcohol use was found 89% higher among cocaine dependents [55]. This might be due to higher increase of reward effects when alcohol and cocaine co-abused compared to either drug self-administered alone, which have been shown in preclinical studies [56–58]. In a study conducted on rats, intravenous injections of cocaine increased alcohol drinking suggesting that cocaine potentiated alcohol seeking [59]. Interestingly, a preclinical study showed a higher genetic susceptibility of the reinforcing effects of cocaine in selectively bred alcohol preferring (P) rats compared to its outbred Wistar rats, which suggests a higher sensitivity of alcoholics to the reinforcing effects of cocaine [60]. Similarly, it has been revealed that genetically predisposed subjects for alcohol dependence have a higher rate to be cocaine dependents [61].

Cocaine co-administered with alcohol can lead to production of cocaethylene, which is more lethal than cocaine itself [62,63]. This cocaethylene can also produce most of the effects that are associated with cocaine [64,65]. Concurrent exposure of alcohol and cocaine may cause more lethality in rats than either drug administered alone, which probably due to the formation of cocaethylene [66]. Interestingly, cocaethylene detection in wastewater has been utilized in recent study as an evidence of co-abuse of cocaine and alcohol in different cities [67]. Cocaethylene levels were found to be significantly higher during weekends compared to weekdays suggesting a higher co-abuse of cocaine and alcohol during weekends [67].

Alcohol has been shown to increase the plasma concentration of cocaine [68]. This is probably mediated through a decrease in the metabolism of cocaine by carboxylesterases, which hydrolyze it to benzoylecgonine and ecgonine methyl ester metabolites [68] (Table 1). Furthermore, it has been demonstrated that alcohol administered with cocaine can lead to increase in cocaethylene concentration in plasma and decrease benzoylecgonine renal excretion (Figure 1) [69]. It is noteworthy that different routes of drug exposure may produce different peak levels of cocaethylene [70]. For example, oral administration is considered the highest in raising cocaethylene concentration in blood as compared to intravenous route [70]. The inhalation route (i.e., smoking) showed the lowest effect on cocaethylene blood concentration compared to oral and IV routes [70]. Furthermore, cocaine and cocaethylene blood concentrations were obtained following concurrent use of cocaine and alcohol [71]. This study revealed that the concentration of cocaine in plasma was found to be increased by 15% after cocaine and alcohol co-exposure. Moreover, 22% of the absorbed cocaine was converted to cocaethylene. Although, cocaine half-life was not altered significantly by ingestion of alcohol, cocaethylene’s half-life was increased in comparison to cocaine’s [71]. Increasing the half-life of cocaethylene might impose serious health problem due to increasing body exposure to its deteriorating toxic effects.

Concurrent exposure to cocaine and alcohol has deleterious effects on cardiovascular and endocrine systems. Co-abuse of cocaine and alcohol was found to increase heart rate, systolic blood pressure, cortisol, and prolactin concentrations (Figure 2) [64,69]. In addition, cerebral blood perfusion was found to be affected by co-exposure to cocaine and alcohol [72,73] (Table 1). It has been shown that cerebral hypo-perfusion was more common among individuals taking cocaine and alcohol compared to individuals taking cocaine or alcohol alone [72,73]. These findings show the significant deleterious effects of the co-abuse of alcohol and cocaine on cardiovascular system that might result in debilitating conditions.

Several tests were performed on intelligence, memory, verbal learning and other aspects of neuropsychological performances to explore the effects of co-abuse of alcohol and cocaine [74,75] (Table 1). The resulting neuropsychological performances were found to be negatively affected by the concurrent intake of cocaine and alcohol compared to either drug administered alone (Figure 3) [74,75]. It has been shown that the sense of pleasure and euphoria increased in co-abuse of alcohol and cocaine and consequently elevated the risk of dependence and toxicity [71,76]. In addition, alcohol was found to significantly potentiate the effect of cocaine in conditioned place preference in rat and invertebrate animal models [56,77]. Moreover, study showed that there is a synergistic effect in self-administration of both alcohol and cocaine in concentrations that did not provoke self-administration to either drug alone [78]. Similarly, a recent study has shown the potentiating effect of cocaine on alcohol seeking and relapse-like alcohol intake in P rats [79]. This might indicate a cross reactivity between alcohol and cocaine on common drug seeking behaviour.

Several studies have shown the involvement of mesolimbic dopaminergic system in reinforcing effects of cocaine [80–83] and alcohol [84,85]. In fact, alcohol and cocaine co-exposure increased extracellular dopamine concentration in the nucleus accumbens, well known brain region involved in the rewarding and reinforcing effects of drugs of abuse [86–89], than either drug administered alone in rats [90] (Table 1). Furthermore, recent findings have demonstrated the critical role of glutamate and its uptake in central brain reward regions in the seeking and reinforcing effects of cocaine [91–93] and alcohol [94–97]. Further studies are needed for investigating the role of glutamatergic system in alcohol and cocaine co-abuse in brain regions involved in rewarding and reinforcing effects of these drugs.

Alternatively, studies have shown the detrimental effects of prenatal exposure to cocaine such as low birth weight, preterm delivery, and decrease in head circumstance [98–101]. However, prenatal co-exposure to cocaine and alcohol has not been well studied despite the findings that more than 85% of women who reported using cocaine during pregnancy, also reported concurrent alcohol use [98]. One recent study, however, has demonstrated a significant interaction in prenatal co-exposure of cocaine and alcohol on cortical thickness in youths prenatally exposed to these drugs [102]. Furthermore, it has been shown that prenatal exposure to alcohol increased the rewarding and reinforcing effects of cocaine in rats (Figure 4) [103].

## Alcohol and nicotine co-abuse

Alcohol and nicotine have serious global health problems. Table 1 summarizes different studies of the effects of alcohol and nicotine co-abuse. Nicotine dependents may have high tendency to be alcohol dependents [104]. It has been reported that more than 80% of chronic alcohol users are also smokers [105–107]. In a preclinical study, rats chronically co-exposed to alcohol and nicotine showed higher nicotine self-administration as compared to drug self-administered alone [108]. Although, it has been suggested that nicotine or alcohol consumed alone may have some beneficial effect at low doses, it is clear that co-abuse of these drugs may have negative effects on human health [109].

Nicotine and alcohol activate the mesocorticolimbic dopaminergic system; there is potential synergistic effect in the increase of dopamine release when the drugs are consumed concurrently [110]. Furthermore, studies showed that alcohol and nicotine co-abuse can lead to increase dopaminergic neuronal firings and dopamine release [111–116] (Table 1). It is suggested that the synergistic effect of these drugs may influence drug reinforcement to each other and predispose smokers to become alcoholics and vice versa [110,117]. Interestingly, an additive effect on dopamine release in the nucleus accumbens shell was found between alcohol and nicotine in rats [116]. This additive effect on dopamine release was inhibited by mecamylamine pre-treatment, a nicotinic receptor antagonist, suggesting the involvement of nicotinic receptors in the reinforcing effects of alcohol. Importantly, alcohol-induced dopaminergic neurons firing in ventral tegmental area were inhibited in mice lacking nicotinic acetylcholine receptors that contain α6 subunit [118]. Moreover, it has been shown that alcohol and nicotine co-abuse can increase the pleasurable effects of each drug [119] (Table 1). This may explain some of the pharmacological mechanisms of action involving the co-abuse of nicotine and alcohol in the modulation of dopamine release (Figure 3).

The risk of developing cancer in general is higher in heavy tobacco smokers and alcohol drinkers [120–123]. In case-controlled clinical studies that were conducted on European and American subjects, alcohol and tobacco smoking elevated the risk of head and neck cancer in patients addicted to both drugs [124]. The exact mechanism of alcohol and nicotine interaction that results in the development of cancer is not well known and remains controversial. Studies have suggested that alcohol and nicotine co-abuse may produce toxic metabolites such as acetaldehyde, which may contribute to cancer development [125,126]. Other studies have suggested that alcohol and nicotine co-abuse promotes the formation of premalignant lesions (Figure 1) [127–129].

The effects of alcohol and nicotine co-abuse on cardiovascular system have been also investigated. Synergistic effects on heart rate and blood pressure were found in healthy human volunteers following alcohol and nicotine exposure [130,131]. Interestingly, the order of self-administering alcohol and nicotine plays a role in their negative interactive effect on cardiovascular system. When self-administration of alcohol was followed by nicotine, a synergistic effect on the increase in left ventricular pressure was revealed, which was alleviated when self-administration of nicotine was followed by alcohol in dogs (Figure 2) [132].

In a study investigated the link between alcohol and nicotine use during pregnancy in more than 14000 previous pregnant mothers, it was found that more than 55% of pregnant alcohol users reported smoking [133]. Alcohol and nicotine exposure during gestational period increased the risk of fetal growth abnormalities more than the exposure to alcohol alone [133–135]. Interestingly, in a study conducted on rats, alcohol and nicotine were co-administered to pregnant rats throughout the gestational period [136]. This study showed that offspring prenatally co-exposed to nicotine and alcohol developed rapid increase in nicotine self-administration as compared to controls (Figure 4) [136].

## Alcohol and MDMA co-abuse

According to 2001–2002 national epidemiologic survey in United States, the prevalence of alcohol use in MDMA users is more than 95% [137]. MDMA and alcohol exposure in adolescent mice induced physiological and behavioural alteration than either drug administered alone [138]. In fact, recent study has shown that the co-abuse of MDMA and alcohol exacerbated cardiac cellular stress and toxicity through augmented activation of cardiac sympathetic system in adolescent mice (Figure 2) [139]. MDMA can induce a rapid release of dopamine and serotonin [140]. High consumption of MDMA may result in depletion of serotonin in the brain, resulting in serious psychological consequences [141–148]. MDMA is usually consumed with many drugs such as amphetamine, cocaine, cannabis, and alcohol. Alcohol and MDMA co-abuse is considered the most popular form of MDMA co-abuse [149–153]. Importantly, mice pre-treated with MDMA were found to consume high amount of alcohol compared to control mice [154] (Table 1). Therefore, mice consumed higher amounts of alcohol to reach to the same reward effect that was normally reached at lower doses of alcohol. This study also found that MDMA impaired the dopaminergic pathway. Furthermore, findings revealed that presynaptic modulation of serotonin release in the hippocampus is affected by exposure to both MDMA and alcohol [155]. This study also showed that long term consumption of MDMA and alcohol caused serotonin depletion. The alteration in the serotonergic system might be associated with the psychopathological disturbances observed in MDMA and alcohol co-abusers [155] (Table 1).

It has been found, in a double blind study conducted on nine healthy human volunteers, that MDMA and alcohol co-abuse induced a longer duration of euphoria and feeling well as compared to drug use alone [149]. Therefore, MDMA and alcohol use together can increase the abuse potential more than abusing alcohol or MDMA alone. In a preclinical study, exposure to alcohol during adolescent age in mice increased the reinforcing effects of MDMA [156]. Moreover, exposure to MDMA and alcohol during adolescence potentiated anxiety measures, impaired learning and memory, and decreased striatal dopamine contents during adult life in mice (Figure 3) [157,158]. Studies have demonstrated an increase in the MDMA plasma concentration by 13% following alcohol intake and a decrease in blood alcohol concentration of about 12% compared to either drug administered alone (Figure 1) [149]. In addition, these studies found that MDMA reversed the subjective sedative effect, which was induced by alcohol consumption.

A recent study aimed to find the effect of MDMA and alcohol co-abuse on learning and memory [159]. In this study, alcohol and MDMA were administered either together or alone to measure their effects on learning and memory in adult mice. Both drugs caused impairment of learning and memory, as the affected mice displayed an imbalance in the interaction of dopamine and serotonin. These findings suggest that the brain in adulthood is very sensitive to MDMA and alcohol damage [159]. However, other study did not demonstrate any additive effect of combining alcohol and MDMA on declarative memory in mice [160] (Figure 3). This might be due to several factors, including the doses used for alcohol and MDMA.

Prenatal exposure to alcohol and MDMA is understudied topic, although pregnant women who reported MDMA use during pregnancy also reported higher alcohol use compared to non MDMA users [161,162]. Importantly, a preclinical study found that prenatal exposure to both alcohol and MDMA impaired working memory, exploratory activity, and neurogenesis in rat’s offspring (Figure 4) [163].

## Conclusion

Alcohol interaction with drugs of abuse is currently not well understood, however, there are studies that demonstrated numerous side effects, which have occurred with drugs co-abuse. The prevalence of concurrent abuse of alcohol with psychostimulants such as METH, cocaine, nicotine, or MDMA is extremely high. This increase in prevalence of co-abuse of alcohol with psychostimulants is most likely due to potentiated effects on euphoria and pleasure as well as decrease detrimental subjective effects of either alcohol or other drugs of abuse. Co-abuse of alcohol with psychostimulants can lead to serious negative consequences on the brain such as decreasing antioxidant enzymes, disrupting learning and memory processes, cerebral hypo-perfusion, neurotransmitters depletion as well as potentiating drug seeking behaviour. Moreover, co-abuse of alcohol and psychostimulants can lead to increase in heart rate, blood pressure, myocardial oxygen consumption and cellular stress as well as increase in the risk of developing different types of cancer.

Alcohol has been shown to increase the blood concentration of different psychostimulants and its active metabolites. It is suggested that the pharmacokinetics of METH, MDMA, cocaine, and nicotine, might be altered when alcohol is consumed concurrently with these drugs. We suggest here that alcohol metabolism and its metabolites may increase the blood concentration of these drugs of abuse, and consequently elevate the risk of toxicity. Importantly, alcohol co-abuse with psychostimulants during pregnancy can impose critical structural and functional damages in the fetal brains. Further studies are needed to investigate possible pharmacodynamics, pharmacokinetics, and neurochemical basis of co-abuse of alcohol and psychostimulants as well as possible therapeutic interventions.

## Figures and Tables

**Table 1:** Aspects and effects of alcohol and psychostimulants interactions.

Drug of Abuse	Aspect of interaction	Effects of interactions
METH	METH metabolism	Alcohol decreased p-hydroxylated metabolites of METH in the urine of METH abusers [33].
		Alcohol increased the levels of METH and its active metabolite, amphetamine, in rats and rabbits [34,35].
	Performance and sleep	Lower detrimental effects on performance and sleep compared to each drug alone [36].
	Euphoria	Increased euphoria in alcohol and methamphetamine co-abuse [36].
	Cardiac effects	Increased myocardial oxygen consumption and cardiac rate [37].
	Prenatal exposure	Damage to striatal region of the brain [41].
	Oxidative stress	Combination caused more impairment of antioxidant enzymes in rats hippocampus and oxidative stress than either drug alone [39].
Cocaine	Cocaine metabolism	Alcohol decreased metabolism of cocaine [68]. Alcohol decreased benzoylecgonine renal excretion, and increased in cocaine and cocaethylene blood concentrations [69].
	Cardiovascular and endocrine systems	Exposure to cocaine and alcohol increased heart rate, systolic blood pressure, cortisol, and prolactin levels [64,69].
	Cerebral blood perfusion	Cerebral hypo-perfusion occurred more in individuals taking cocaine and alcohol than in individuals taking cocaine or alcohol alone [72,73]
	Neurobehavioral performances	Negatively affected by concurrent intake of cocaine and alcohol compared to either drug alone [74,75].
	Mesocorticolimbic dopamine system	Increased extracellular dopamine concentration than either drug alone in nucleus accumbens in rats [90]
		Sense of pleasure and euphoria were found to be improved [71].
Nicotine	Drug reinforcement	Rats have established self-administration and place preference to combination of alcohol and cocaine in concentrations that did not provoke reinforcement to either drug alone [56,78]. Cocaine potentiated alcohol seeking [59,79]
	Mesocorticolimbic dopamine system	Increased in dopaminergic neuron firings and dopamine release in an additive mechanism [111–116].
	Pleasure and drug seeking	Increased in the pleasurable effects of each drug [119]. Rats self-administered nicotine more than rats received chronic exposure to either drug alone [108].
	Cardiovascular system	Additive effect on heart rate and blood pressure was found in healthy human volunteers [130,131]. Synergistic increase in left ventricular pressure in dogs [132].
	Cancer	Increase in the risk of developing esophageal cancer [120–123].
	Prenatal exposure	Showed a multiplicative effect in increasing the risk of head and neck cancer in human [124].
		Increased the risk of fetal growth restrictions in human [133–135]. Offspring developed rapid nicotine self-administration and at a higher level in rats [136].
MDMA	Cardiovascular system	Exacerbated cardiac cellular stress and toxicity through augmented activation of cardiac sympathetic system in adolescent mice [139].
	Blood level	MDMA plasma concentration increased following alcohol intake [149].
	Drug reinforcement	MDMA and alcohol induce a longer duration of euphoria [149].
		Exposure to alcohol during adolescent age in mice increased the reinforcing effects of MDMA [156].
	Sedation	MDMA reversed the sedative effect induced by alcohol consumption [149].
	Learning and memory	Administration of alcohol and MDMA exhibited learning and memory impairments [159]
	Dopamine reward effect	MDMA impaired dopaminergic reward pathway, leading to increase alcohol consumption [154].
	Psychopathological effect	Long term consumption of MDMA and alcohol can lead to serotonin depletion and cause psychopathological changes [155].
	Prenatal exposure	Impaired working memory, exploratory activity, and neurogenesis in rats offspring [163].
